# Baculovirus-mediated endostatin and angiostatin activation of autophagy through the AMPK/AKT/mTOR pathway inhibits angiogenesis in hepatocellular carcinoma

**DOI:** 10.1515/biol-2022-0914

**Published:** 2024-07-29

**Authors:** Tingting Wei, Jiajie Cheng, Yonggan Ji, Xue Cao, Shuqin Ding, Quanxia Liu, Zhisheng Wang

**Affiliations:** Department of Oncology, General Hospital of Ningxia Medical University, Ningxia Hui Autonomous Region, Yinchuan, 750001, P.R. China; School of Pharmacy, Ningxia Medical University, Ningxia Hui Autonomous Region, Yinchuan, 750004, P. R. China; School of Inspection, Ningxia Medical University, Ningxia Hui Autonomous Region, Yinchuan, 750004, P.R. China

**Keywords:** hepatocellular carcinoma, baculovirus, endostatin-angiostatin, anti-angiogenesis, autophagy

## Abstract

Hepatocellular carcinoma (HCC) is a highly vascularized carcinoma, and targeting its neovascularization represents an effective therapeutic approach. Our previous study demonstrated that the baculovirus-mediated endostatin and angiostatin fusion protein (BDS-hEA) effectively inhibits the angiogenesis of vascular endothelial cells and the growth of HCC tumors. However, the mechanism underlying its anti-angiogenic effect remains unclear. Increasing evidence suggests that autophagy has a significant impact on the function of vascular endothelial cells and response to cancer therapy. Hence, the objective of this research was to investigate the correlation between BDS-hEA-induced angiogenesis inhibition and autophagy, along with potential regulatory mechanisms. Our results demonstrated that BDS-hEA induced autophagy in EA.hy926 cells, as evidenced by the increasing number of autophagosomes and reactive oxygen species, accompanied by an upregulation of Beclin-1, LC3-II/LC3-I, and p62 protein expression. Suppression of autophagy using 3-methyladenine attenuated the functions of BDS-hEA-induced EA.hy926 cells, including the viability, proliferation, invasion, migration, and angiogenesis. Moreover, BDS-hEA induced autophagy by downregulating the expression of CD31, VEGF, and VEGFR2, as well as phosphorylated protein kinase B (p-AKT) and phosphorylated mammalian target of rapamycin (p-mTOR), while concurrently upregulating phosphorylated AMP-activated protein kinase (p-AMPK). The *in vivo* results further indicated that inhibition of autophagy by chloroquine significantly impeded the ability of BDS-hEA to suppress HCC tumor growth in mice. Mechanistically, BDS-hEA prominently facilitated autophagic apoptosis in tumor tissues and decreased the levels of ki67, CD31, VEGF, MMP-9, p-AKT, and p-mTOR while simultaneously enhancing the p-AMPK expression. In conclusion, our findings suggest that BDS-hEA induces autophagy as a cytotoxic response by modulating the AMPK/AKT/mTOR signaling pathway, thereby exerting anti-angiogenic effects against HCC.

## 1 Introduction

Hepatocellular carcinoma (HCC) is a prevalent human malignant tumor, constituting about 80% of all cases of primary liver cancer [1]. The current therapeutic approaches continue to encounter challenges attributed to the highly aggressive metastatic propensity of HCC [1]. The growth and metastasis of HCC are primarily driven by angiogenesis, resulting in a high incidence of hematogenous metastases and exceptionally elevated mortality [2]. Consequently, inhibition of neovascularization has become one of the effective strategies to impede tumor progression and dissemination.

Currently, numerous endogenous anti-angiogenic agents, such as endostatin, tumstatin, and angiostatin, have been extensively investigated and their anti-tumor angiogenesis effects were confirmed [3]. Nevertheless, the therapeutic efficiency of utilizing these protein-based inhibitors remains limited due to their short half-life and the potential for development of drug resistance with long-term administration [4]. In recent years, the sustained expression of foreign genes through gene therapy appears to be a promising approach for overcoming these limitations. Previously, we developed a bivalent baculovirus vector that not only resists complement system inactivation but also continuously expresses foreign genes. Expression of the endostatin (collagen α1-chain XVIII C-terminal hydrolytic fragment) and angiostatin (amino-terminal proteolytic fragment of plasmin) fusion protein using this vector (BacSC-DAF-SB-T2ChEA, abbreviated as BDS-hEA) exhibited enhanced and sustained inhibition of vascular endothelial cell angiogenesis, proliferation, and migration, along with significant suppression of HCC tumor growth in mice [5]. Moreover, when combined with gemcitabine treatment, the synergistic therapeutic effect was more pronounced than a single application [6]. Nevertheless, the precise mechanism underlying the anti-tumor angiogenic effect of BDS-hEA remains unclear.

Autophagy, a self-protective mechanism developed by cells during long-term evolution, is a process in which damaged substances undergo self-degradation through lysosomes in response to external interference or disruption of cellular homeostasis [7]. The process of autophagy is an intricate biological phenomenon, involving multiple signaling pathways in its regulation. For example, the mammalian target of rapamycin (mTOR), a cellular nutrient and energy level sensor, negatively regulates autophagy, while protein kinase B (AKT) and AMP-activated protein kinase (AMPK), acting as upstream regulators, positively and negatively regulate mTOR levels, respectively [8]. Emerging findings indicate a potential association between autophagy and the angiogenic activity observed in different types of cancers [9]. However, there appears to be contradictory evidence regarding the involvement of autophagy in angiogenesis. For instance, Sun et al. confirmed that arsenic trioxide effectively inhibited angiogenesis and induced autophagy in human umbilical vein endothelial cells (HUVECs), and inhibition of autophagy significantly reversed their anti-angiogenic effect [10]. Interestingly, Xue et al. reported that lenvatinib could induce autophagy in thyroid cancer cells through the PI3K/AKT/mTOR pathway, and autophagy suppression enhanced the cytotoxicity and anti-angiogenic capacity of lenvatinib [11]. Nevertheless, the precise role and regulatory mechanism of autophagy underlying BDS-hEA-induced anti-angiogenesis have not been completely clarified.

In this research, we explored the relationship between BDS-hEA-induced inhibition of angiogenesis and autophagy, elucidating their underlying molecular mechanisms. Our findings demonstrate that BDS-hEA induces autophagy as a cytotoxic response by modulating the AMPK/AKT/mTOR pathway. These findings highlight the potential of targeting autophagic response to enhance the therapeutic efficacy of angiogenesis inhibitors.

## 2 Materials and methods

### 2.1 Reagents

Antibodies against VEGFR2 and VEGF were obtained from Santa Cruz (California, USA); antibody against LC3B was purchased from Abmart (Shanghai, China); antibodies against Beclin-1, p62, CD31, Ki67, AKT, p-AKT, mTOR, p-mTOR, AMPK, p-AMPK, and HRP-labeled rabbit/mouse IgG were procured from Abcam (Cambridge, USA); antibody against MMP-9 was obtained from Proteintech (Wuhan, China); and antibodies against β-actin and FITC-labeled rabbit/mouse IgG were procured from ZSGB-BIO (Beijing, China). TUNEL red fluorescence *in situ* apoptosis detection kit was purchased from Vazyme (Nanjing, China). Reactive oxygen species (ROS) detection kit was obtained from Solarbio (Beijing, China). 3-Methyladenine (3-MA) was obtained from APExBIO (Houston, USA). Chloroquine (CQ) was purchased from MCE (New Jersey, USA).

### 2.2 Viruses and cells

The construction and transduction methods of recombinant baculovirus BDS-hEA have been described previously [5]. EA.hy926 cells (human umbilical vein endothelial fusion cell line) and HepG2 cells (human hepatocellular carcinoma cell line) were grown in DMEM (Sigma, USA) containing 10% fetal bovine serum (FBS, Gibco, USA) and 1% penicillin/streptomycin solution (Gibco, USA) in a 5% CO_2_ incubator with a temperature of 37°C.

### 2.3 Monodansylcadaverine (MDC) and mGFP-LC3-adenovirus staining

EA.hy926 cells with or without 3-MA (4 mM) in the medium were incubated in 12-well plates (7 × 10^4^ cells/well) overnight. Subsequently, the cells were transduced with BDS-hEA for 48 h at different multiplicity of infection values (MOIs) and then stained with 200 μL of MDC solution (Solarbio, Beijing, China) in dark at room temperature for 30 min. Alternatively, the EA.hy926 cells were exposed to mGFP-LC3 adenovirus (HANBIO, Shanghai, China) at a concentration of 10 MOI for 24 h prior to transduction with BDS-hEA for another 48 h. Finally, the formation and visualization of autophagosomes were observed under an inverted fluorescence microscope.

### 2.4 ROS detection

EA.hy926 cells were grown in 12-well plates (7 × 10^4^ cells/well) overnight and then tansduced with BDS-hEA at MOI values of 100 and 400 for 48 h. Subsequently, each well was treated with 200 μL of diluted DCFH-DA fluorescent probe (10 μmol/L), followed by incubation at 37°C for 20 min. The cells were then observed and documented under an inverted fluorescence microscope.

### 2.5 Cell viability assay

EA.hy926 cells with or without 3-MA (4 mM) in the medium were incubated in 96-well plates (7 × 10^3^ cells/well) overnight, followed by transduction with BDS-hEA (MOI 400) for 48 h. Afterward, fresh DMEM supplemented with 10% CCK-8 solution (Beyotime, Shanghai, China) was introduced into each well and incubated for 30 min at 37°C. The microplate reader (Multiskan GO, Thermo, USA) was then utilized to measure the optical density (OD) value at a wavelength of 450 nm for every group that had five technical replicates. Cell viability (%) was calculated as follows: (OD_treatment_ − OD_blank_)/(OD_control_ − OD_blank_) × 100%.

### 2.6 Colony formation assay

EA.hy926 cells with or without 3-MA (4 mM) were transduced with BDS-hEA (MOI: 400) for 48 h, and then the cells in each group were trypsinized and inoculated into 6-well plates (700 cells/well). The cells were cultured for approximately 2 weeks until visible colonies became apparent. Following that, the colonies were fixed using 4% paraformaldehyde, dyed with 0.1% crystal violet, and photographed by an inverted microscope.

### 2.7 Cell wound healing assay

EA.hy926 cells with or without 3-MA (4 mM) were grown in six-well plates (7 × 10^5^ cells/well) overnight. Subsequently, the cells were transduced with BDS-hEA (MOI: 400) for 48 h. When the cells had completely covered the bottom surface of each well, a gentle scraping was performed utilizing a 200 μL pipette tip. Following that, the medium was changed to DMEM supplemented with 1% FBS. Immediate and subsequent photographs were captured at a time interval of 24 h post-incubation. The migration ratio was then calculated by comparing the average migrated area with the initial scratched area, utilizing ImageJ software (NIH, USA).

### 2.8 Cell invasion assay

EA.hy926 cells with or without 3-MA were transduced with BDS-hEA (MOI 400) for 48 h, and then the cells were resuspended (2 × 10^4^ cells/well) in FBS-free DMEM and seeded in the upper chamber (precoated with a matrix solution containing 0.5 mg/mL from Corning, NY, USA) of a 24-well transwell plate with a pore diameter of 8 μM. The lower chamber contained DMEM enriched with 10% FBS. After 24 h, the upper chamber non-invasive cells were scraped off with a cotton swab, while the invasive cells situated beneath the membrane were immobilized using a 4% paraformaldehyde and dyed utilizing a 0.1% crystal violet solution. Finally, images were taken using an inverted microscope.

### 2.9 Tubule formation assay

EA.hy926 cells with or without 3-MA were transduced with BDS-hEA (MOI 400) for 48 h, and then the cells were resuspended and seeded in precoated matrigel (50 μL/well) in 96-well plates (3 × 10^4^ cells/well) for 4 h at 37°C. After that, the formation of tubule networks was observed by an inverted fluorescence microscope (100×). Quantification was conducted by utilizing ImageJ software to calculate the cumulative length of master segments in five fields that were randomly chosen.

### 2.10 Western blot assay

EA.hy926 cells with or without 3-MA were cultured in a 10 cm cell culture dish and subsequently transduced with BDS-hEA at different MOI values for 48 h. Afterward, the cells were harvested, lysed, and the protein concentration was determined using the BCA kit (KeyGen, Nanjing, China). Equivalent quantities of protein were separated by 10–12% SDS-PAGE gels and then moved onto a PVDF membrane (Millipore, MA, USA). Subsequently, the bands were blocked using skim milk for a duration of 1 h and incubated overnight at 4°C with primary antibodies. Following that, the bands were exposed to secondary antibodies for 1 h at room temperature. Next, the bands were exposed to an enhanced chemiluminescence (ECL) solution (Thermo, USA) for 1 min in darkness before being subjected to analysis and quantification utilizing the gel imaging system (Bio-Rad, USA) and ImageJ software (NIH, USA), respectively.

### 2.11 HCC xenograft model in mice

Twenty male BALB/c nude mice (4–6 weeks, Weitong Lihua, Beijing, China) were housed in a standardized SPF-grade barrier facility. The animal experiment adhered to the ethical guidelines that were approved by the Ethics and Welfare Committee of Ningxia Medical University (IACUC-NYLAC-2021-135). HepG2-EGFP and luciferase cells (EGFP = enhanced green fluorescent protein), which stably expressed EGFP and luciferase through lenti-CMV-EGFP and luciferase infection, were introduced into each mouse’s right dorsal region via subcutaneous injection using 100 μL of PBS containing 2 × 10^6^ cells. After the tumor volume grew to around 100 mm^3^, a random allocation was made to divide the mice into four groups with five animals per group for subsequent injections as follows: control group (intratumoral injection of an equal volume of PBS in a 7-day interval four times); BDS-hEA group (intratumoral injection of 1 × 10^8^ pfu/mouse in a 7-day interval four times); CQ group (intraperitoneal injection of 50 mg/kg/mouse every 2 days); and BDS-hEA + CQ group (identical dose and frequency as previously). The measurement of tumor volume was conducted at 3-day intervals using a dial caliper, and the tumor growth status was real-time-monitored every 7 days using a small animal live imaging system (IVIS LUMINA III, USA). On day 28 post-administration, all mice were euthanized using a CO_2_ inhalation euthanasia device (SMQ-II-Q, Shanghai, China), and tumor tissues were stripped for the following tests. (Only the doser has access to information about animal grouping throughout the entire process.)


**Ethical approval:** The research related to animal use complied with all the relevant national regulations and institutional policies for the care and use of animals.

### 2.12 Immunohistochemistry staining

Tumor tissues were treated with 4% paraformaldehyde, followed by embedding in paraffin. Subsequently, the paraffin sections were processed for dewaxing, hydration, antigen retrieval, and sealing according to the immunohistochemistry kit (ZSGB-BIO, Beijing, China). The tissue sections were then subjected to overnight incubation at 4°C with each of the primary antibodies Beclin-1, LC3B, p62, p-AKT, p-mTOR, and p-AMPK. Afterward, the sections were exposed to secondary antibodies conjugated with HRP for 1 h at ambient temperature. Diaminobenzidine (DAB) was utilized as a chromogen to visualize the antigen–antibody complexes, and hematoxylin was employed as a counterstain. The photographs were captured using a fluorescence microscope (Ni-U, Nikon, Japan), then five photos were randomly selected, and the integrated optical density (IOD)/area was measured utilizing Image-Pro Plus 6.0 software (Media Cybernetics, MD, USA) to evaluate the positive expression rate of each section.

### 2.13 Immunofluorescence and TUNEL staining

The fresh tumor specimens were embedded in OCT solution and subsequently prepared as frozen sections. Antigen retrieval and sealing procedures were then conducted according to the instructions provided in the immunofluorescence kit (ZSGB-BIO, Beijing, China). The sections were subsequently subjected to overnight incubation at 4°C with each of the primary antibodies CD31, VEGF, Ki67, and MMP-9. Following that, FITC-conjugated corresponding species secondary antibodies were applied to incubate at room temperature for 1 h, followed by counterstaining with DAPI in darkness for 10 min. The TUNEL assay was conducted following the standard protocol provided by Vazyme (Nanjing, China) to detect tissue apoptosis. The images were photographed and analyzed as described above.

### 2.14 Statistical analysis

The data obtained from this study were subject to one-way analysis of variance using SPSS 26.0 software (IBM, NY, USA). The results were based on a minimum of three independent experiments. All the experiments were repeated at least three times. Statistical significance was determined at *p* < 0.05, *p* < 0.01, and *p* < 0.001.

## 3 Results

### 3.1 BDS-hEA induces autophagy in vascular endothelial cells

**Figure 1:**
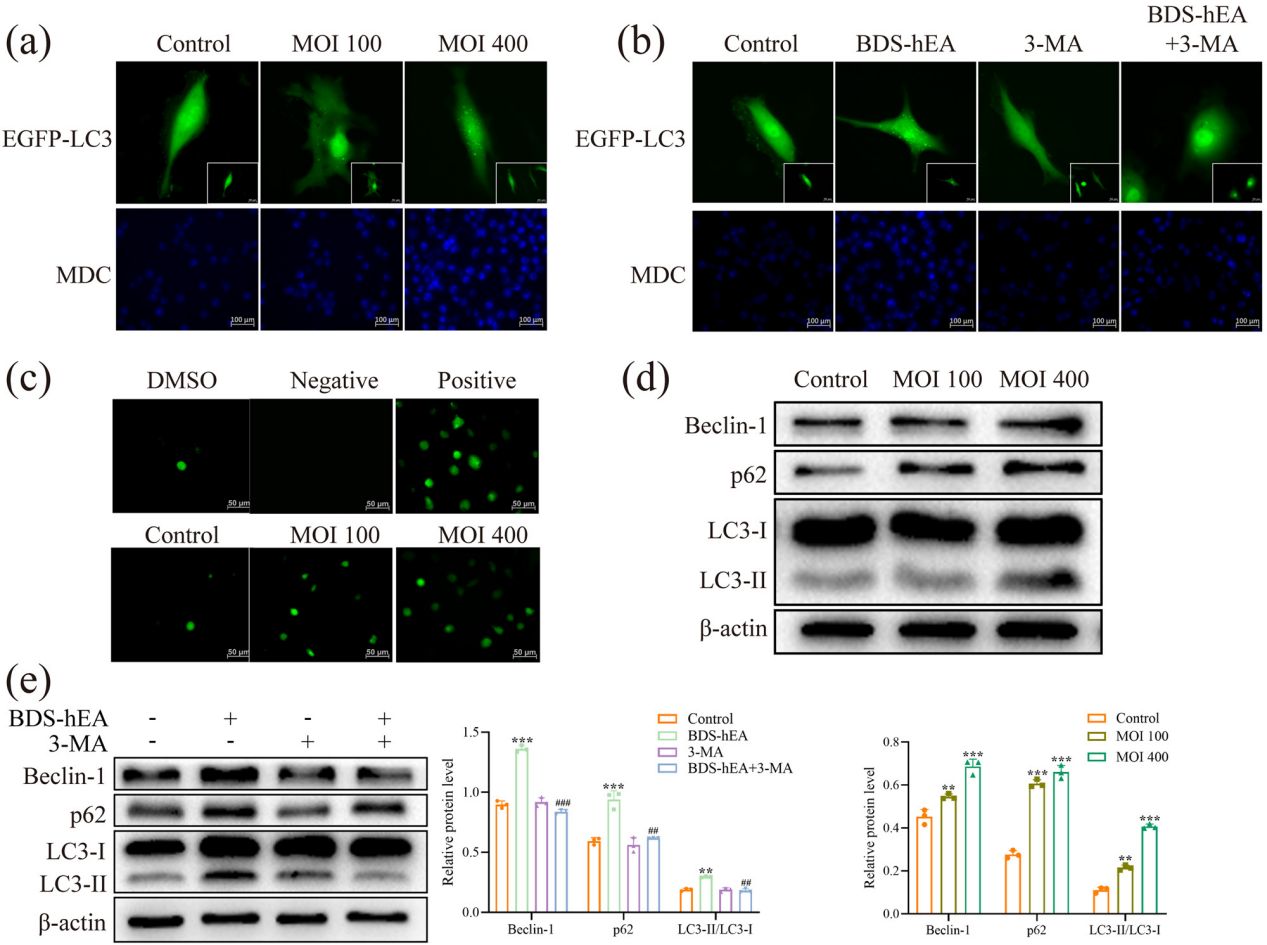
BDS-hEA induces autophagy in vascular endothelial cells. The EA.hy926 cells were transduced with BDS-hEA for 48 h with or without 3-MA. EGFP-LC3-adenovirus and MDC staining were used to detect the morphology of autophagy without (a) or with 3-MA (b). (c) ROS was detected by the DCFH-DA fluorescent probe. The Beclin-1, p62, LC3-I, and LC3-II proteins were analyzed by western blot without (d) or with 3-MA (e), with β-actin as a loading control. All the experiments were repeated at least three times. **p < 0.01 and ***p < 0.001 vs control group. ^##^ *p* < 0.01 and ^###^ *p* < 0.001 vs BDS-hEA group.

Previous research has demonstrated the significant inhibitory effects of BDS-hEA on the viability and tubule formation of vascular endothelial cells [5]. However, the underlying mechanism behind this phenomenon remains unclear. Extensive research works have suggested a strong correlation between tumor angiogenesis and autophagy [9]. To investigate whether BDS-hEA can induce autophagy in vascular endothelial cells, we examined the changes in autophagic morphology by tracking the number of autophagosomes through staining with EGFP-LC3-adenovirus and MDC. Figure 1a demonstrates a dose-dependent rise in the number of EGFP-LC3-adenovirus-labeled green spots and MDC-labeled blue cells within the BDS-hEA group, as compared to the control group. After pretreatment with 3-MA to inhibit autophagosome formation, followed by transduction with BDS-hEA (MOI 400), a noticeable decrease in the quantity of fluorescent cells was found after combined treatment compared to BDS-hEA alone in EA.hy926 cells (Figure 1b). Studies have indicated that the initiation of autophagy potentially lead to the buildup of intracellular ROS [12]. Hence, an investigation was further conducted to assess the intracellular levels of ROS using the fluorescent probe 2ʹ7ʹ-dichlorodihydrofluorescein diacetate (DCFH-DA). The finding revealed a significant dose-dependent increase in ROS levels within the BDS-hEA group in comparison with the control group (Figure 1c). Additionally, the Beclin-1, p62, and LC3-II/LC3-I proteins related to autophagy were further assessed through western blot. The results depicted in Figure 1d indicated that there was a gradual increase in the expression of aforementioned proteins in the BDS-hEA group with increasing doses. However, when combined with 3-MA, a significant trend of protein reversal was observed in comparison to the BDS-hEA group (Figure 1e). These findings collectively confirm that BDS-hEA has the capability to trigger autophagy in EA.hy926 cells.

### 3.2 Blocking autophagy impairs the functionality of vascular endothelial cells induced by BDS-hEA

**Figure 2:**
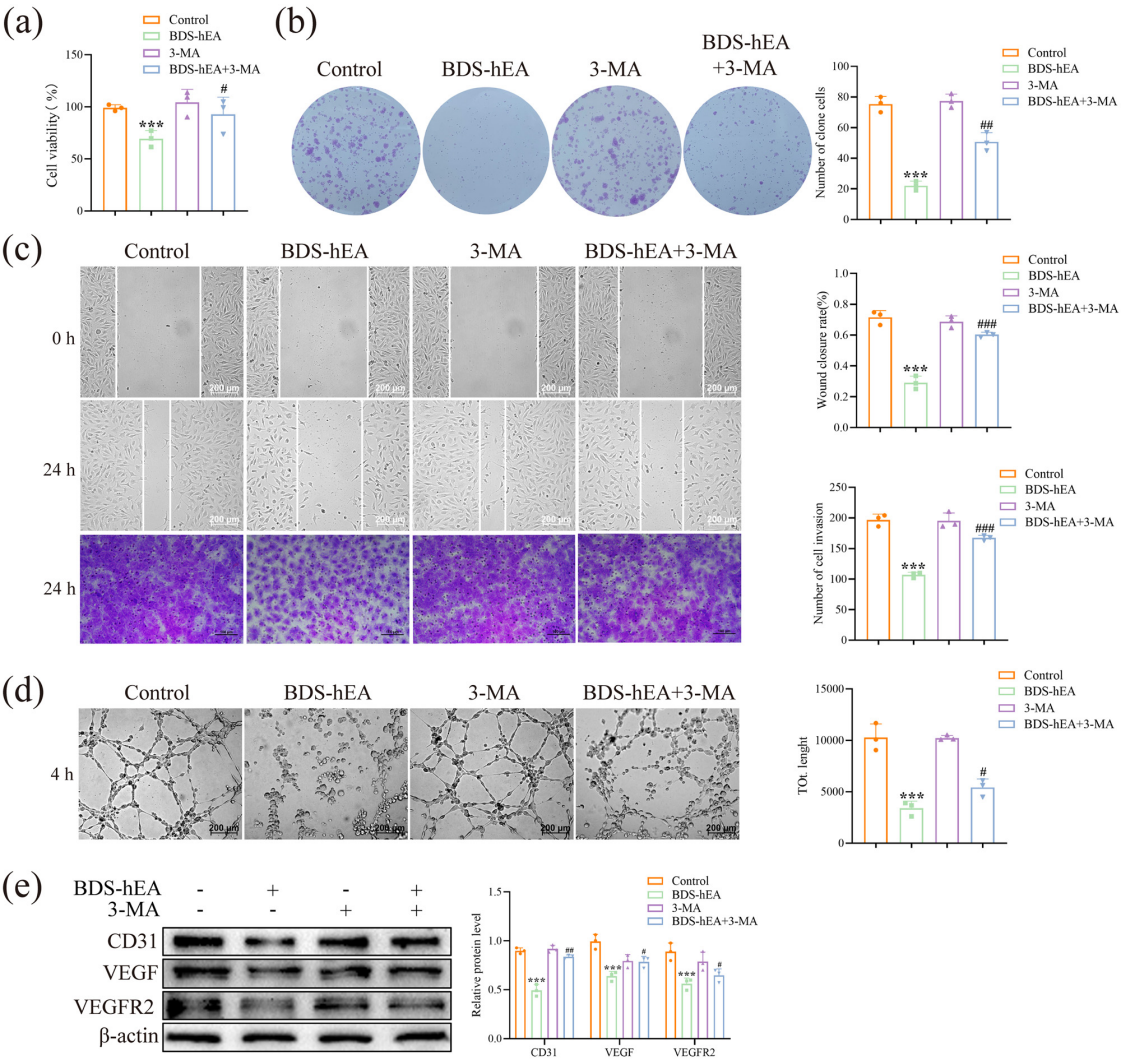
Blocking autophagy impairs the functionality of vascular endothelial cells induced by BDS-hEA. The EA.hy926 cells were transduced with BDS-hEA (MOI, 400) for 48 h with or without 3-MA. (a) Cell viability was evaluated by the CCK-8 assay. (b) Cell proliferation ability was assessed by the colony formation assay. (c) Cell migratory and invasive capacities were evaluated by scratch and transwell assays. (d) Vascularization ability was measured by the tubule formation assay. (e) Detection of angiogenesis-related proteins after 3-MA intervention. The data from three repeated experiments were used for the statistical analysis. ***p < 0.001 vs control group. ^#^ *p* < 0.05, ^*#*#^ *p* < 0.01, and ^###^ *p* < 0.001 vs BDS-hEA group.

To further investigate the effect of BDS-hEA-induced autophagy on the function of vascular endothelial cells, we assessed the impact of 3-MA-mediated autophagy inhibition on BDS-hEA-induced EA.hy926 cell proliferation, invasion, and migration, as well as tubule formation and angiogenesis-related protein expression alterations. The results from CCK-8 and clone formation assays showed that there is a significant enhancement in the cell viability and proliferation ability within the BDS-hEA + 3-MA-treated group compared to the BDS-hEA group (Figure 2a and b). Furthermore, BDS-hEA + 3-MA significantly attenuated the migration, invasion, and angiogenic capacity of EA.hy926 cells treated with BDS-hEA, as demonstrated by the cell scratch, invasion, and tubule formation assays (Figure 2c and d). Additionally, we investigated the alterations in proteins associated with angiogenesis and found that BDS-hEA significantly suppressed the expression of CD31, VEGF, and VEGFR2 proteins. When combined with 3-MA, there was a significant reversal trend in comparison with the BDS-hEA group (Figure 2e). In combination, these results suggest that the inhibition of autophagy hinders the growth, invasion, migration, and angiogenesis in BDS-hEA-induced vascular endothelial cells.

### 3.3 BDS-hEA induces autophagy in vascular endothelial cells through modulation of the AMPK/AKT/mTOR signaling pathway

**Figure 3:**
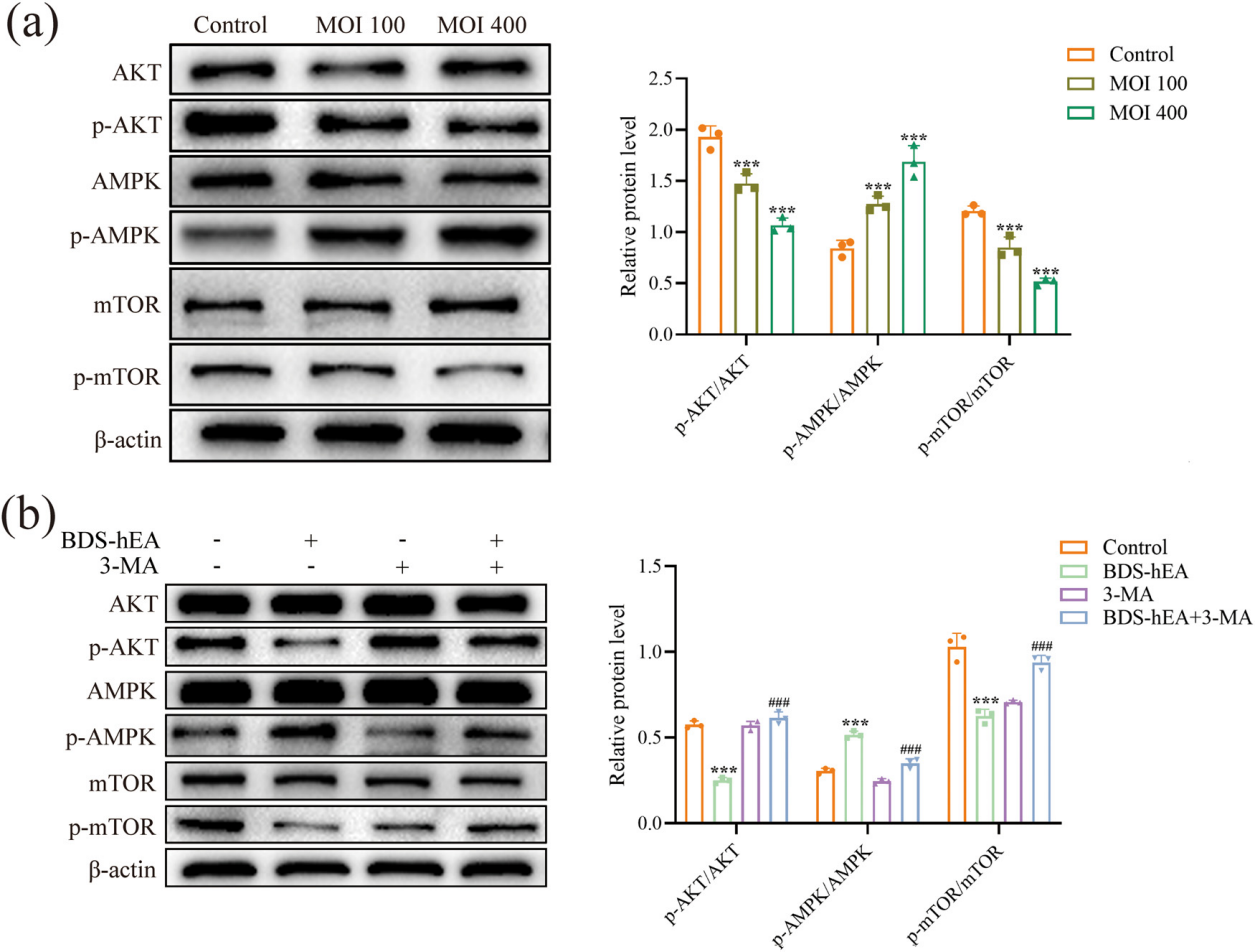
BDS-hEA induces autophagy in vascular endothelial cells through the modulation of the AMPK/AKT/mTOR signaling pathway. The pathway-related proteins were detected by western blot in EA.hy926 cells treated with BDS-hEA (MOI 100 or 400) for 48 h without (a) or with 3-MA (b), with β-actin as a loading control. Three repeated experiments were used for analysis. ***p < 0.001 vs control group; ^###^ *p* < 0.001 vs BDS-hEA group.

Numerous studies have confirmed that AMPK and AKT/mTOR pathways are implicated in the regulation of autophagy [13]. Therefore, we performed the western blot assay to investigate the potential involvement of this cascade in BDS-hEA-treated EA.hy926 cells. As depicted in Figure 3a, with the escalation of BDS-hEA dosage, an evident rise in the expression level of p-AMPK/AMPK was observed, whereas there was a notable decrease in p-AKT/AKT and p-mTOR/mTOR levels when compared to those in the control group. When 3-MA was added, the aforementioned protein levels were reversed in comparison to those observed in the BDS-hEA group alone (Figure 3b). Overall, BDS-hEA induces the autophagy of EA.hy926 cells by activating AMPK and suppressing AKT/mTOR pathways.

### 3.4 BDS-hEA inhibits the growth of HCC by inducing autophagy in tumor tissues

**Figure 4:**
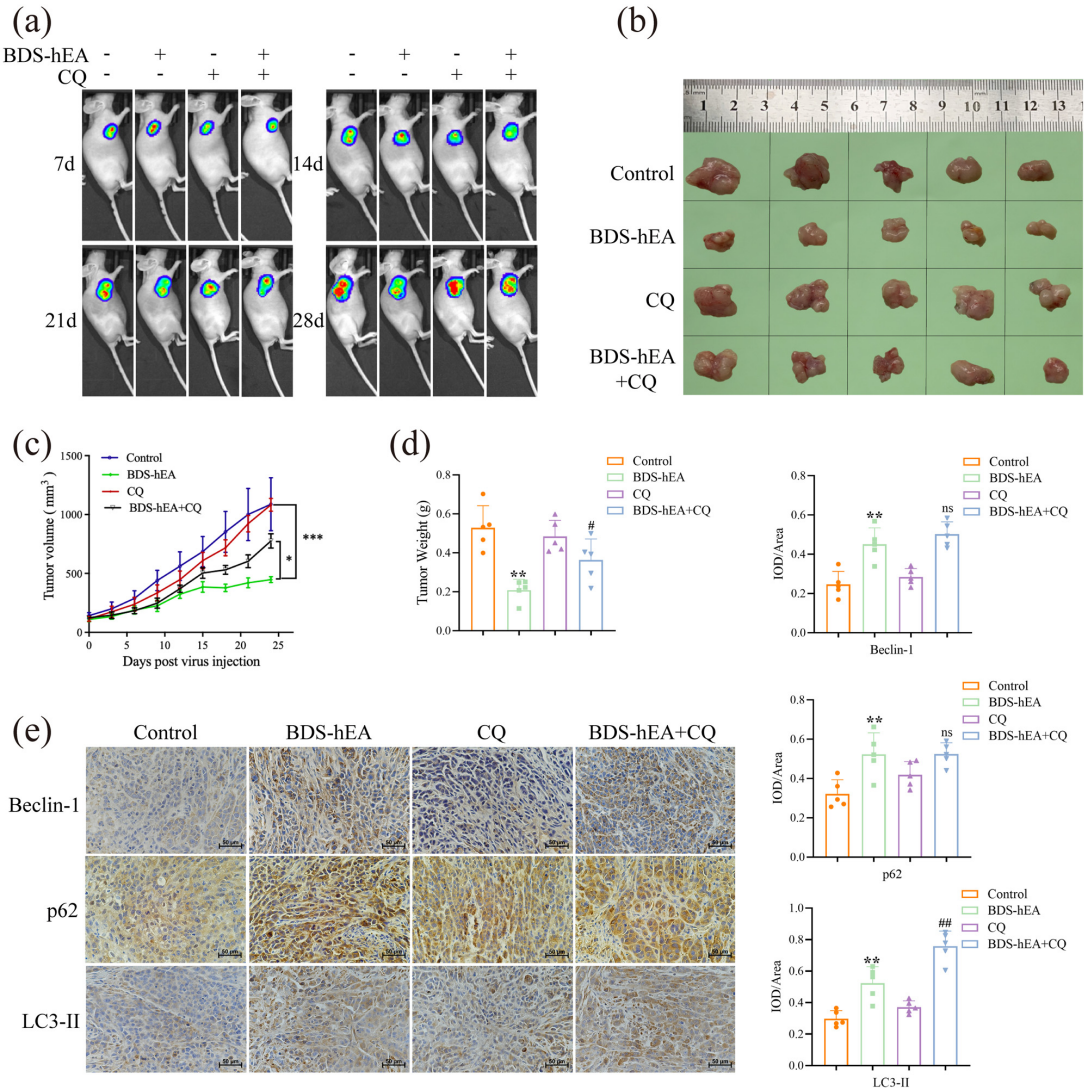
BDS-hEA inhibits the growth of HCC by inducing autophagy in tumor tissues. (a) Tumor growth status was real-time-monitored using a small animal live imaging system. (b) Tumor anatomy of mice. (c) Tumor volume statistical chart. (d) Tumor weight statistical chart. (e) Beclin-1, p62, and LC3-II expression levels were determined by immunohistochemical analysis. *p < 0.05, **p < 0.01, and ***p < 0.001 vs control group. ^#^ *p* < 0.05 and ^##^ *p* < 0.01 vs BDS-hEA group. ns means no significance vs BDS-hEA group.

Our previous study has demonstrated that BDS-hEA can effectively inhibit the growth of HCC xenograft tumors in mice [5], but the relationship between its inhibitory effect and autophagy remains unclear. Therefore, we evaluated the inhibitory effect of BDS-hEA on HCC by creating a xenograft model of nude mice, before and after intervention with the autophagy inhibitor CQ. The results revealed a gradual enhancement in the fluorescence intensity of control tumors over time, whereas the BDS-hEA group exhibited significantly diminished fluorescence compared with the control group. Conversely, the BDS-hEA + CQ group showed an augmented fluorescence intensity compared to the BDS-hEA group, with a more pronounced reversal trend, particularly at 28 days post-administration (Figure 4a). Similar findings were also confirmed that the BDS-hEA group exhibited significant reductions in the tumor size, volume, and weight when compared remaining three groups. Notably, the BDS-hEA + CQ group showed a significant counteraction of these parameters in comparison to the BDS-hEA alone group (Figure 4b–d).

To further validate the induction of autophagy by BDS-hEA in tumor tissues, we conducted immunohistochemical analysis to assess the protein levels of p62, Beclin-1, and LC3-II. As shown in Figure 4e, the BDS-hEA group showed a significant increase in the expression levels of p62, Beclin-1, and LC3-II (yellow or brown particles appeared in the cytoplasm or cell membrane) when in comparison to the control group. When combined with CQ, there was a further upregulation observed in these proteins. This phenomenon possibly ascribes to the inhibitory effect of CQ on late-stage autophagy, which prevents the fusion between autophagosomes and lysosomes, thereby hindering the degradation process of these proteins. Altogether, these results indicate that activation of autophagy is responsible for the inhibitory effects of BDS-hEA on transplanted HCC tumors.

### 3.5 BDS-hEA inhibits tumor proliferation, migration, and angiogenesis by inducing autophagic apoptosis in tumor tissues

**Figure 5:**
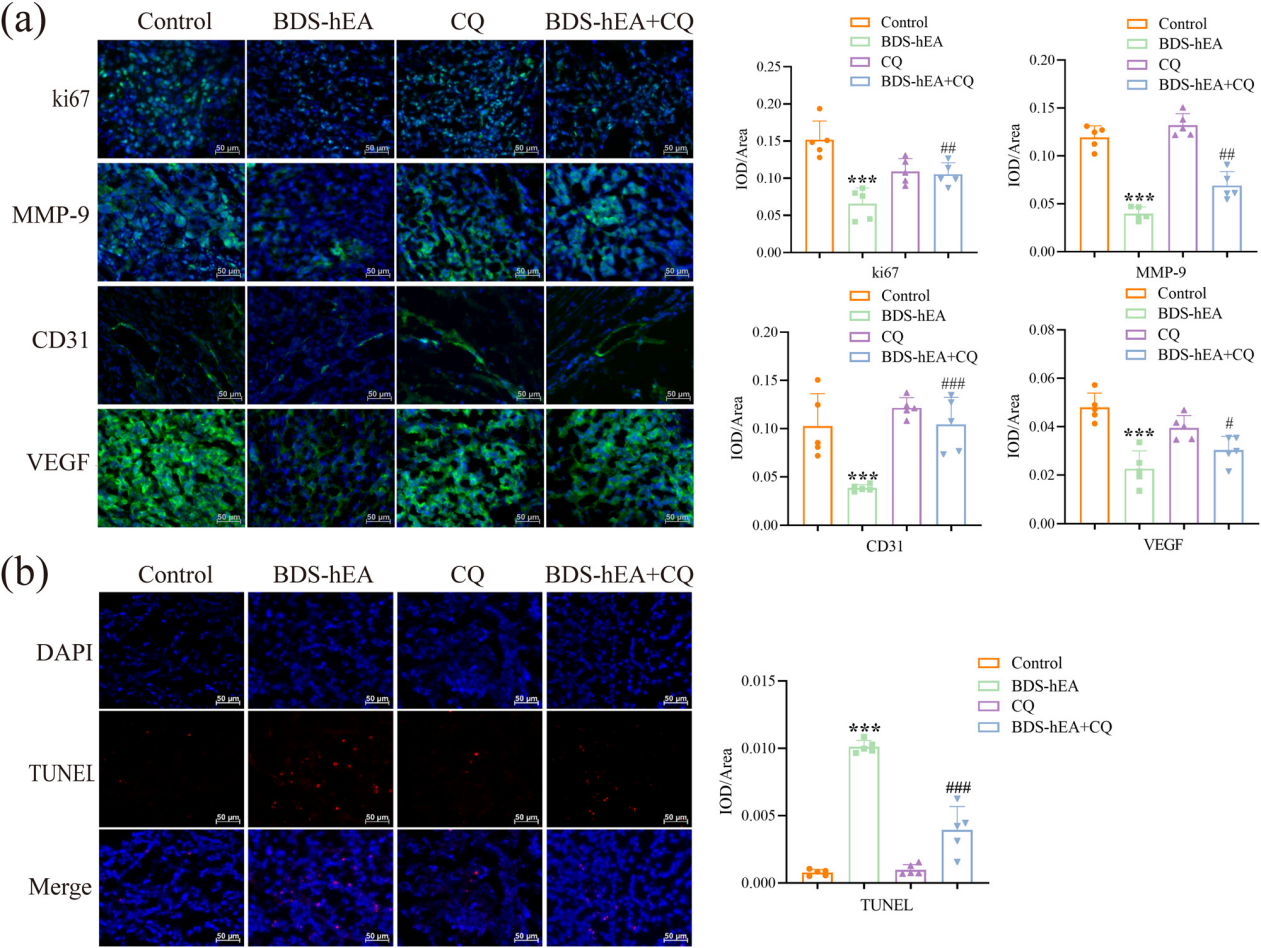
BDS-hEA inhibits tumor proliferation, migration, and angiogenesis by inducing autophagic apoptosis in tumor tissues. (a) Immunofluorescence staining was used to analyze the expression changes of ki67, MMP-9, CD31, and VEGF proteins. (b) Tumor tissue apoptosis was assessed using TUNEL staining. All results were repeated three times. Five fields were randomly selected for statistics. ***p < 0.001 vs control group; ^#^ *p* < 0.05, ^##^ *p* < 0.01, and ^###^ *p* < 0.001 vs BDS-hEA group.

In order to investigate the potential mechanism of BDS-hEA inhibiting HCC tumor growth, immunofluorescence staining was employed to analyze the expression changes of tumor proliferation (ki67), migration (MMP-9), and angiogenesis (CD31 and VEGF). Figure 5a shows that BDS-hEA significantly downregulated the expression levels of ki67, MMP-9, CD31, and VEGF in comparison to the control group. However, it is noteworthy that the expression levels of these proteins were found to be significantly higher in the BDS-hEA + CQ group than those observed in the BDS-hEA group alone. The activation of autophagy was found to be concomitant with the apoptosis of tissue cells [14]. To further investigate their interrelationship, we assessed the extent of apoptosis in tumor tissues using TUNEL staining. As illustrated in Figure 5b, BDS-hEA significantly increased the population of red fluorescent apoptotic cells, whereas a decrease was noted in the BDS-hEA + CQ group when compared with the BDS-hEA alone. In all, the above results suggest that BDS-hEA exerts a tumor-suppressor role by inducing autophagy-dependent apoptosis to inhibit the expression of proteins associated with tumor proliferation, migration, and angiogenesis.

### 3.6 BDS-hEA induces autophagy in tumor tissues through modulation of the AMPK/AKT/mTOR signaling pathway

**Figure 6:**
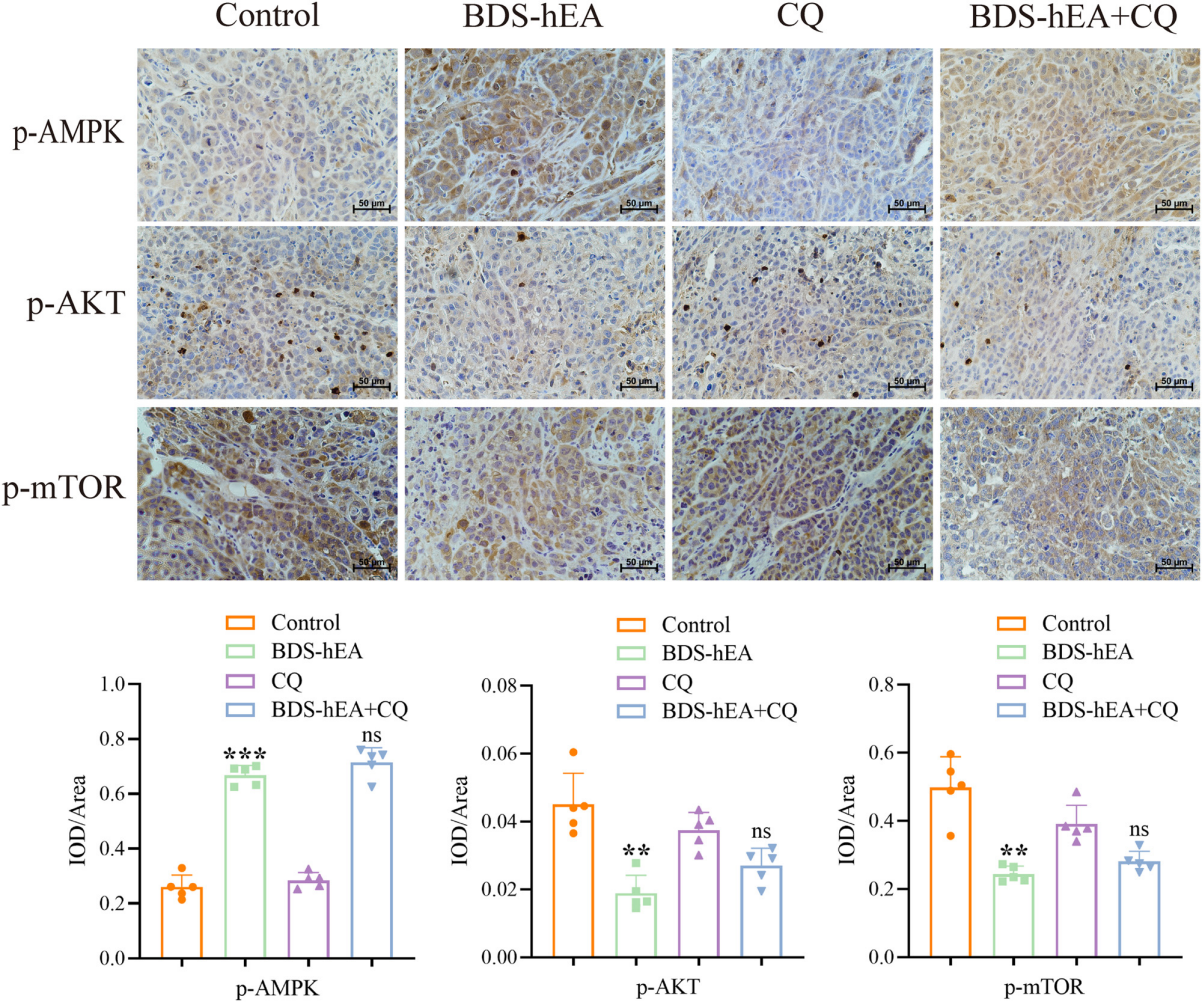
BDS-hEA induces autophagy in tumor tissues through the modulation of the AMPK/AKT/mTOR signaling pathway. The immunohistochemistry assay was performed to assess the levels of p-AMPK, p-AKT, and p-mTOR in tumor tissues. All results were repeated three times. Five fields were randomly selected for statistics. **p < 0.01 and ***p < 0.001 vs control group; ns means no significance vs BDS-hEA group.

To gain deeper insights into the molecular regulatory mechanism of autophagy induced by BDS-hEA *in vivo*, the immunohistochemistry assay was conducted to assess the levels of p-AMPK, p-AKT, and p-mTOR in tumor tissues. The results indicated a significant rise in the rate of positive expression of p-AMPK, accompanied by a notable reduction in p-AKT and p-mTOR levels within the BDS-hEA group compared to the control group (Figure 6). However, when combined with CQ, changes in the expression of these proteins were not statistically significant compared to the BDS-hEA group, which may be due to the fact that CQ blocked the downstream autophagic flow but did not affect the occurrence of upstream autophagy. Collectively, our data indicate that BDS-hEA regulates autophagy *in vivo* by activating AMPK and inhibiting AKT/mTOR signaling.

## 4 Discussion

Angiogenesis is a multifaceted biological process involving the activation, proliferation, migration, and tube formation of endothelial cells, ultimately leading to the development of new blood vessels [15]. Neovascularization is crucial for the provision of essential nutrients and oxygen to tissues, thereby playing an indispensable role in facilitating tumor progression and metastasis [16]. Inhibition of angiogenesis has emerged as a promising therapeutic strategy against tumors. Endostatin is a 20 kDa C-terminal hydrolytic fragment derived from the XVIII collagen α 1-chain, which has been extensively studied as one of the endogenous angiogenesis inhibitors [17]. The protein drug Endostar, a recombinant form of human endostatin, received approval from China’s State Food and Drug Administration in 2005 for the treatment of non-small cell lung cancer [18], highlighting the potential efficacy of endostatin in cancer therapy. Angiostatin, the initial Kringle domain among four domains found in a 38 kDa internal proteolytic fragment of plasminogen, has been acknowledged as a potent endogenous inhibitor of angiogenesis and extensively demonstrated for its anti-tumor efficacy [19]. The primary obstacle hindering the future application of these drugs in clinical trials, however, lies in their limited therapeutic efficacy and short half-life [3]. In the past few years, tumor gene therapy has emerged as a valuable therapeutic strategy, complementing conventional treatments such as surgery, chemotherapy, and radiotherapy. Gene therapy offers distinct advantages such as sustained and localized expression of therapeutic gene products, low cost, and minimal side effects, making it a promising alternative to conventional protein drugs for cancer treatment [20]. However, the delivery efficiency and safety of gene therapy still need to be further improved.

The establishment of an efficient and secure delivery system is the foundation of gene therapy. Baculovirus, being an insect-specific virus, possesses several notable advantages over currently used viral vectors such as adenovirus, adeno-associated virus, retrovirus, and lentivirus. These advantages encompass its non-pathogenicity to humans, non-immunogenicity, ability to transduce both dividing and non-dividing cells, as well as lower the cell toxicity in mammalian cells [21,22]. Nevertheless, the application of baculovirus is constrained due to its susceptibility to complement system inactivation and the transient expression of exogenous genes [23]. In our previous work, we successfully developed a bivalent baculovirus gene delivery system by displaying the decay accelerating factor (DAF) on the viral surface to protect against complement attack while employing the Sleeping Beauty transposon system for efficient integration of exogenous genes into the host chromosome [5] and confirmed that the hybrid viruses had no significant inhibitory effect on the vitality and proliferation of vascular endothelial cells, indicating that our constructed viral vector is safe. We further utilized this system to express the fusion protein of human endothelin and angiostatin (BDS-hEA), confirming that the recombinant protein has significant inhibitory effects on tumor angiogenesis and growth in mouse HCC [5]. When combined with gemcitabine, it exhibited even stronger inhibition of tumor growth. Additionally, we found that BDS-hEA had less hepatotoxicity compared to gemcitabine, indicating that gene therapy mediated by baculovirus exhibits weaker toxic effects than chemotherapy drugs [6]. Given the excellent anti-tumor efficacy and safety of BDS-hEA, this study aims to further elucidate its anti-angiogenic mechanism.

Emerging evidence reveals a crucial role of autophagy in endothelial cells, the primary constituents of the vasculature responsible for delivering nutrients and oxygen to tissues. Furthermore, recent studies suggest that autophagy governs pathological angiogenesis, a characteristic feature of solid tumors [24]. The hyperproliferative nature of tumors readily lead to hypoxia and malnutrition within the tumor microenvironment. Under these adverse circumstances, heightened autophagy in blood vessels is emerging as a critical mechanism. Autophagy is a dynamic process encompassing the initiation, formation, maturation, and degradation of autophagosomes [25]. Multiple autophagy-related proteins actively participate in this intricate pathway. For instance, Beclin-1 plays a pivotal role in both the initiation and maturation stages of autophagosome biogenesis, while LC3-I undergoes transformation into LC3-II during the entire process of autophagosome formation [26]. Additionally, p62, an adaptor protein that negatively regulates autophagy, primarily participates in proteolytic degradation through the autophagy–lysosome system and the ubiquitin–proteasome system [27]. Therefore, increased levels of Beclin-1 and LC3-II, as well as decreased levels of p62, indicate the presence of autophagic activity. Our study observed an increased formation of autophagosomes, accompanied by upregulated expression of Beclin-1, LC3-II/LC3-I, and p62 (Figure 1a and d). Suppression of autophagy *in vitro* using 3-MA effectively reversed the aforementioned indicators (Figure 1b and e), indicating that BDS-hEA has the ability to trigger autophagy in vascular endothelial cells. Furthermore, the transplantation of mouse HCC further confirmed the capacity of BDS-hEA to trigger autophagy, as demonstrated by the upregulation of Beclin-1, LC3-II, and p62 proteins (Figure 4e). It is noteworthy that the activation of autophagy by BDS-hEA does not lead to a decrease in the level of p62, which serves as a marker for assessing the autophagic flux. When BDS-hEA is combined with the autophagy inhibitor CQ, there is a further increase in the levels of Beclin-1, LC3-II, and p62. This observation suggests that BDS-hEA impedes the autophagy flux, resulting in the accumulation of autophagosomes without subsequent degradation. Our findings suggest that BDS-hEA exerts effects similar to CQ, and further investigation is needed to determine whether it disrupts lysosomal membrane integrity and hinders fusion between autophagosomes and lysosomes. Recent research works have demonstrated that p62 can independently regulate oxidative stress and inflammation apart from its role in autophagy degradation [28]. Clinical investigations have revealed a correlation between the accumulation of p62 and unfavorable outcomes in patients diagnosed with HCC [29]. This may potentially explain the limited efficacy of anti-angiogenic drugs. Currently, we are exploring the relationship between the aberrant expression of p62 and the resistance to BDS-hEA treatment. Furthermore, ROS was found to be a key signaling molecule essential for regulating endothelial cell proliferation, migration, and angiogenesis while also playing an important role in mediating cellular autophagy [30]. Meng et al. found that fascaplysin can inhibit angiogenesis and increase ROS levels by activating autophagy, and blocking ROS production attenuates the fascaplysin-induced autophagic response [31]. Our findings provide additional evidence that BDS-hEA effectively enhanced ROS levels in a dose-dependent pattern (Figure 1c). Collectively, our results provide novel evidence that BDS-hEA has the ability to induce autophagy in vascular endothelial cells and HCC tumors.

The relationship between autophagy and angiogenesis remains inconclusive in various studies due to the autophagy’s dual nature. For instance, rapamycin-induced autophagy has been found to facilitate pro-angiogenic effects in the HUVECs [32]. Conversely, another study demonstrated that mebendazole induces autophagy in endothelial cells, leading to an anti-angiogenic effect [33]. Our investigation demonstrated that the functions of BDS-hEA-treated EA.hy926 cells were counteracted upon inhibition of autophagy, as evidenced by the reversal in cell viability, proliferation, invasion, migration, and angiogenesis as well as the expression levels of VEGF, VEGFR2, and CD31 (Figure 2). Furthermore, the tumor growth-suppressive properties of BDS-hEA in mice were compromised when autophagy was inhibited, as demonstrated by the results of Figure 4a–d. This inhibitory effect was ascribed to the promotion of apoptosis in tumor tissues and the restraint of proteins associated with tumor proliferation (ki67), metastasis (MMP-9), and angiogenesis (CD31 and VEGF) expression (Figure 5). Collectively, these findings underscore the role of autophagy as a suppressor in inhibiting HCC tumor growth mediated by BDS-hEA.

Studies have reported that the angiogenesis inhibitor bevacizumab can exacerbate hypoxia and nutritional deficiencies within the tumor microenvironment [34]. As a cellular sensor, mTOR exerts positive regulation on cellular nutrient uptake, energy metabolism, and stress response signaling [35]. Under stressful conditions such as nutrient deprivation and hypoxia, the AKT/mTOR axis is suppressed, thereby inducing autophagy and degradation of cellular components for energy acquisition [36]. Additionally, the AKT/mTOR pathway is also crucially involved in the regulation of neovascularization, and inhibiting its activity effectively suppresses angiogenesis [37]. For example, Wu et al. reported that the combination of Endostar and anti-PD-1 significantly inhibited lung cancer angiogenesis in mice by inhibiting the PI3K/AKT/mTOR signaling pathway to activate autophagy [18]. AMPK serves as another crucial intracellular sensor that plays a key role in negatively regulating cellular energy levels and inducing autophagy in response to conditions of oxidative stress and energy deprivation [38]. Upon stimuli, AMPK can activate autophagy by directly phosphorylating and activating ULK1 or indirectly activating ULK1 with TSC2-mediated mTOR inhibition [39]. Our results provided evidence supporting the induction of autophagy by BDS-hEA both *in vitro* (Figure 3) and *in vivo* (Figure 6), through the activation of AMPK and inhibition of AKT/mTOR signaling pathways.

## 5 Conclusions

In summary, our investigation revealed that BDS-hEA induces autophagy in vascular endothelial cells and HCC tumors by activating AMPK and suppressing AKT/mTOR signaling pathways and that autophagy inhibition blocks the BDS-hEA-induced functions of vascular endothelial cell proliferation, metastasis, and angiogenesis as well as HCC tumor growth. These findings provide a theoretical foundation for developing targeted molecules against the autophagy pathway to enhance the therapeutic efficacy of antiangiogenic drugs.
